# Supplementary material for: Validity of self-reported weight and height among female young adults in the United Arab Emirates
Source: PLoS One. 2024 Apr 18;19(4):e0302439. doi: 10.1371/journal.pone.0302439 (PMC11025931; doi:10.1371/journal.pone.0302439)
Supplement: Attachment — Submitted filename: Response to Author 1.docx [file pone.0302439.s001.docx]

8^th^ November 2023

REF: PONE-D-23-35645

Title: Validity of self-reported weight and height among female young adults in the United Arab Emirates
Dr. Dalia Haroun

In response to the response in relation to the discrepancy in the ethics submission. Kindly note that ethical approval was obtained for the study: “Caffeine and energy drink consumption, perceived benefits and related health effects amongst UAE university students”. This study has already been published in the Arab Journal of Nutrition and Exercise Volume 1, Issue no. 1, DOI 10.18502/ajne.v1i3.1230.

Our current study titled: ‘“Validity of self-reported weight and height among female young adults in the United Arab Emirates”, submitted to Plos One, used data from the existing database. However, all statistical analyses, and the drafting and reviewing of the manuscript was carried out by the authors listed on the submitted manuscript. Rola Al Ghali was not involved in the current manuscript.

Please advise if there is a still a need to revise the authorship according to the above.

Best wishes,

Dr. Dalia Haroun
